# Editorial: Structure-Related Intrinsic Electrical States and Firing Patterns of Neurons With Active Dendrites

**DOI:** 10.3389/fncel.2018.00229

**Published:** 2018-08-22

**Authors:** Sergey M. Korogod

**Affiliations:** Department of Molecular Biophysics, O. O. Bogomoletz Institute of Physiology, National Academy of Sciences of Ukraine, Kiev, Ukraine

**Keywords:** dendrites, voltage-gated conductances, excitability, intrinsic firing patterns, synaptic and dendritic integration, electric coupling, computer modeling

Activity of neurons embedded in networks is an inseparable composition of intrinsic and evoked processes. Prevalence of either component depends on the neuron's function (e.g., signal pacemaker vs. transmitter) and state (e.g., low vs. high depolarization states). Complex firing patterns of a neuron are conventionally attributed to complex spatial-temporal organization of inputs received from the network-mates via synapses, in vast majority dendritic. However, these views require revisiting with account of active properties of the dendrites. Structural features of the arborization inevitably impact on electrical states of its constituting parts at different levels of organization, from branches and sub-trees to ion channels. This Research Topic aimed at bringing together contributions of researches from different domains and gaining deeper insight into the nature of neuronal intrinsic firing patterns. Being cross-listed in Frontiers in Cellular Neuroscience and Frontiers in Computational Neuroscience, it contains 22 articles (14 and 8 in the respective journal specialties, respectively) including 14 original research articles, 4 reviews; 1 mini review, 1 methods, and 2 hypothesis and theory articles.

## Historical perspectives of studies of structure-related intrinsic neuronal activity

Llinás who pioneered in discovery of active membrane properties of neuronal dendrites and putting forward the notion the intrinsic activity of neurons, provides a historical perspective of studies, which flagged beginning of modification of the reflex viewpoint of brain function, as the global neuroscience paradigm, toward one, in which sensory input modulates rather than dictates brain function. The author explains how unique firing signatures of different type neurons are related to their specific sets of voltage-gated ion channels, dendritic in particular, and notes that complex intrinsic properties allow neurons to function either as relay systems, or as oscillators and/or resonators.

Bower describes his view of the history, achievements and merits of the first “community model,” a Purkinje neuron model with detailed morphology and appropriate active conductances of the dendrites. The author emphasizes on importance of using such models for testing, interpreting, and predicting experimental data rather than for demonstrating the plausibility of a particular idea and illustrates implementations of this approach in their “community model” of Purkinje cell.

## Dendritic origins of firing patterns in neurons

Alford and Alpert review dendritic mechanisms of synaptic integration in neurons forming the spinal central pattern generator in lamprey. These mechanisms transform an unpatterned glutamatergic input into a patterned, rhythmic output that is a feature of the spinal network. Dendritic origin of the intrinsic oscillatory activity is defined by the interplay of Ca^2+^ entry through NMDA-type glutamatergic channels with contribution from voltage-gated Ca^2+^ channels and outward current through Ca^2+^-sensitive K^+^ channels producing in-phase oscillations of intracellular Ca^2+^ and the membrane potential in the dendrites.

Magnani et al. address specific oscillatory and firing properties of stellate cells in layer II of the medial entorhinal cortex. Using the quadratic sinusoidal analysis the authors compare characteristics of subthreshold membrane potential oscillations and supra-threshold firing of action potentials (APs) generated in response to multi-sinusoidal current stimulation. The quadratic responses were likely dominated by the dendrites and contained frequencies that were not present in the input signal and the characteristics of the subthreshold oscillations at resonance frequencies near the threshold were similar to those of the supra-threshold spike trains.

Based on the analysis of somatic and dendritic plateau properties observed in spiny neurons of amygdala, striatum, and cortex Oikonomou et al. describe their hypothesis stating that the somatic voltage upstates are determined by dendritic plateau potentials. This view is supported in experiments using voltage-sensitive dye imaging, which reported rising of the somatic plateau after the onset of the dendritic voltage transient and collapsing with the breakdown of the dendritic plateau depolarization. It is hypothesized that dendritic plateau potentials underlay detection and transformation of coherent network activity into a ubiquitous neuronal upstate.

Psarrou et al. investigated the effects of the basal dendrites morphology on the firing behavior on models of reconstructed pyramidal neurons in layer V of rat prefrontal cortex. Earlier studies revealed in these cells characteristic firing patterns: regular spiking (RS), intrinsic bursting (IB), and repetitive oscillatory bursting. Variation of dendritic geometry and distribution of ion conductances allowed the authors to derive pattern-predictive structural characteristics. The RS- or IB-generating cells were best discriminated by the total length, volume, and branch number, regardless of the distribution of conductances in basal trees.

Tran-Van-Minh et al. review the biophysical determinants of linear, sublinear, and supralinear effects of multiple co-activated synapses contacting active neuronal dendrites. The authors highlight the interplay of dendritic morphology and channels, spiking threshold and distribution of synaptic inputs. Sublinear relations are favored by the combination of thin dendritic diameter and low expression of voltage-gated channels, whereas thick dendrites expressing voltage-gated channels of inward current favor supralinear relations, from boosting synaptic depolarization to regenerative dendritic spikes.

## Firing patterns in normally developing and degenerating neurons

The geometry, expression and properties of membrane ion channel of neuronal dendrites are subject to changes during normal development and neurodegenerative disease. Some of these aspects are addressed in the following contributions.

Durand et al. explored mouse lumbar motoneurons in isolated spinal cord at a postnatal age of P3-P9 just before mice weigh-bear and walk. The authors characterized the reconstructed dendritic geometry and firing patterns evoked by somatically applied depolarizing currents, particularly of triangular ascending-descending time course (ramps). A transient type pattern was firing during the ascending phase of the current. It was observed in about 40% of cells between P3 and P5 and tended to disappear with age. Linear and clockwise hysteresis firing patterns dominated at P6–P7 age. Prolonged sustained and counterclockwise hysteresis (mature) firing patterns emerged at P8–P9 age and likely were related to maturation of dendritic L-type Ca^2+^ channels. Hence, it is P8–P9 age, when the electrical properties of mouse motoneurons rapidly change to provide the mature motor behaviors.

Dhupia et al. on a model of reconstructed CA1 pyramidal neuron studied the role of geometry of atrophied dendrites in electrical responsiveness of the dendritic tree with distributed hyperpolarization-activated h-channels. The atrophy was mimicked by pruning outer branches. Based on analysis of responses evoked by sinusoidal currents of constant amplitude and linearly increasing frequency, the authors conclude that, in the presence of an h-channel gradient, atrophied neurons respond to incoming inputs and transfer signals across the dendritic tree more efficiently, have significantly diminished spatial gradients of input resistance and local/transfer impedance than those in unpruned cell.

## Functional compartmentalization of dendrites and somato-dendritic coupling

Firing patterns of spinal motoneurons containing channels of persistent inward current (PIC) in the dendritic membrane were explored by Kim and Heckman on a two-compartment model. The authors analyzed model responses to application of triangular current depending on the somato-dendritic electrical coupling, dendritic location and activation of PIC conductances. A variation of PIC activation parameters mimicking neuromodulatory effects of brain stem systems led to narrowing the structure-dependent coupling resistance range, in which the model generated nonlinear (hysteretic) firing patterns. Outside the range, the firing mode became linear irrespectively of PIC location. It is concluded that neuromodulation by the brainstem systems may play a role in switching the motoneurons between linear and non-linear firing modes.

Manuel et al. investigated a two-compartment model of lumbar motoneuron expressing L-type Ca^2+^ conductance and Ca^2+^ -sensitive K^+^ conductance responsible for afterhyperpolarization (AHP) and having a strong electrical coupling of the somatic and dendritic compartments. The cells with different somato-denritic distribution of those conductances were stimulated by triangular ramp currents to determine conditions for a counterclockwise hysteresis of firing frequency-to-current relation associated with the motoneuron bistability. This occurred when L-type conductance in proximal dendrite or soma was co-expressed with and counterbalanced by the AHP conductance. The authors conclude that for the motoneuron firing pattern the dynamical interaction between the L-type and AHP currents is as fundamental as the segregation of the L-type current in dendrites.

Simões-de-Souza et al. developed computational models of three classes of the olfactory bulb granule cells with distinct distributions of spines along their active reconstructed dendrites and investigated how each class integrate synaptic inputs. The classes were defined by the regions, to which their dendrites were confined: the whole external plexiform layer for class I, and lower or upper 1/2 to 1/3 of this layer for class II or III, respectively. Independently of the location of the stimuli and the dendritic tree morphology, the AP always originated in the terminal dendrites and required different quantities of spines to be activated in each dendritic region. The authors conclude that these model predictions might have important computational implications in the context of functioning of olfactory bulb circuits.

Yang et al. studied response properties of CA1 pyramidal neurons in acute brain slices employing the 3D digital holographic photolysis to uncage glutamate at multiple dendritic sites. The somatic responses were integrated supra-linearly or sub-linearly if the stimulation sites were, respectively, clustered on a single dendrite or distributed across multiple dendrites. Such difference was observed for oblique and basal dendrites, but not for the tuft dendrites responding linearly to both types of stimulation. Multi-branch integration occurring at oblique and basal dendrites allows somatic AP firing to follow the driving stimuli over a significantly wider frequency range than in case of single branch integration. However, multi-branch integration requires greater input strength to drive the somatic APs. These data show that integration of such driving signals in a single dendrite is fundamentally different from that in multiple dendrites.

In a study on models of reconstructed CA1 pyramidal cells, Ferrante and Ascoli analyzed how synaptically evoked spiking in these neurons exhibiting higher or lower excitability is regulated by different feedforward inhibition (FFI) GABAergic pathways. The pathways mediated by fast-spiking, perisomatic-targeting basket cells and regular-spiking, dendritic-targeting bistratified cells were stimulated separately or jointly at different strengths. Bistratified interneurons affected low-, but not high-excitable pyramidal cells; whereas basket cells affected both pyramidal cell types similarly. Selective FFI produced by bistratified and basket cells alone modulated respectively, threshold and gain of pyramidal cell firing. Simultaneous FFI via both pathways acting synergistically enlarged the dynamic range of response. The authors conclude that their results provide experimentally testable hypotheses of the differential function of those interneurons.

Iannella and Launey used a biophysically detailed model of a reconstructed neocortical layer 2/3 pyramidal cell to investigate the effect of changes in parameters of the spike timing-dependent plasticity (STDP) of dendritic synapses on the formation of the so-called “dendritic mosaic” composed of clusters of synapses with similar efficacies. The mosaic formation depended on the balance between potentiation and depression, mean presynaptic firing rate and, crucially, the dendritic morphology. Any imbalance led to degradation of such cluster organization. The authors suggest that, synaptic plasticity favors the formation of clustered efficacy engrams.

## Tools for studies of dendritic and axonal processes

Du et al. describe an approach to the reduction of models of neurons possessing weakly excitable large dendritic trees and the strongly excitable small spike initiation zone. It is illustrated on an example of the lobula giant movement detector neuron of the locust. An initial 879-compartment model was transformed by decoupling its branches, reducing separately active and quasi-active branches, re-coupling these two reduced components into a resulting model. The latter being faster retained the full integrative qualities of the original two-order larger model as follows from close similarity of these two models responses to similar stimuli.

Slice preparations are common in electrophysiological studies of neurons and identification of their processes as axon or dendrites in the ongoing experiment is not trivial. Petersen et al. describe a new method allowing reliable identification of axon initial segment (IS) and dendrites by timing of averaged somatic spike and local field potential (LFP) recorded near a targeted neurite. Informative is the timing of the negative LFP event relative to the spike threshold calculated as the first positive peak on the third derivative of the LFP: the event starting before or after reaching the somatic spike threshold indicated location of the LFP electrode near axon IS or dendrite origin, respectively.

Biophysical properties of synaptic receptor channels are important for determining of both efficacy of synaptic transmission and activation of dendritic voltage-gated channels underlying active properties of dendrites. Stepanyuk et al. describe a new method using a maximum likelihood approach to non-stationary fluctuation analysis that allows to estimate a number of synaptic transmission parameters from a small set of postsynaptic current recordings. The method is illustrated on examples of processing of simulated macroscopic synaptic currents, from which the pre-defined parameters of synaptic receptor channels were accurately retrieved.

Singh and Zald describe a new form of dendrite-to-soma transfer function employing separation of slow and fast components of the dendritic electrical events. On an example of analysis of postsynaptic signal transfer along dendrites possessing non-linear NMDA-type conductance, the authors show that their linear “hook” function, being a computational cost-efficient alternative to sigmoid transfer functions, correctly reproduces saturation and linear behaviors for large and small inputs, respectively.

## Fine temporal structure of firing patterns

Mrówczyński et al. in their mini-review discuss occurrence and functional significance of the doublets of the APs frequently observed at the onset of contractions of mammalian motor units during recruitment to strong or fast movements. The authors draw attention to the duration of the AHP, which follows the APs, results from activation of corresponding potassium conductance and significantly influences firing rate in both slow and fast motoneurons.

Mlinar et al. examined spiking activity in a large number of genetically identified serotonergic neurons of the dorsal raphe nucleus (DRN) in slices. They found wide homogeneous distribution of firing rates suggesting that, in terms of intrinsic firing properties, the DRN serotonergic neurons represent a single cellular population. The majority of neurons exhibited regular, pacemaker-like activity with the spiking regularity proportional to the firing rate. In a small subset of neurons, the firing rate exhibited low frequency oscillations. The observed transitions between regular and oscillatory firing suggested that the oscillatory firing mode is an alternative to regular firing in serotonergic neurons.

Cho et al. explored fine temporal structure of firing APs evoked in nociceptive cutaneous C-fibers by application of noxious chemical stimuli and related the firing patterns with pain behavior. They extracted groups of three consecutive spikes (spikelets) and analyzed their duration and within-group inter-spike intervals. The analysis revealed substance-specific patterns: continuous firing for KCl, single or multiple bursts for capsaicin, and repeated short bursts (chattering) for GABA. The authors suggested that information about the agonist chemicals may be encoded by C-afferents in specific temporal patterns, which, via different temporal summation of postsynaptic responses, may influence the pain sensation.

## Author contributions

The author confirms being the sole contributor of this work and approved it for publication.

### Conflict of interest statement

The author declares that the research was conducted in the absence of any commercial or financial relationships that could be construed as a potential conflict of interest.

